# Supplementary material for: Learning curve and surgical outcome of robotic assisted colorectal surgery with ERAS program
Source: Sci Rep. 2022 Nov 29;12:20566. doi: 10.1038/s41598-022-24665-w (PMC9709162; doi:10.1038/s41598-022-24665-w)
Supplement: Supplementary file 1 — Supplementary Information 1. [file 41598_2022_24665_MOESM1_ESM.zip › LSC Raw data-Ñ[▒K/127/H_ñw╝╨░O▒Kñσ.pdf]

正式摘要(電子簽章：00000218847042)

W85 / W85 ☐英文標題(注意:無英文姓名-請補病人基本檔!) ☐【讀取】憑證

臺中榮民總醫院

出院病歷摘要

轉入號 : 轉出號 :

[redacted]

(8)流水編號	
(9)入院日期	110/06/05 (2021)
(10)轉科日期	
(11)出院日期	110/06/11 (2021) 住院天數計 6 日

診斷

- 入院 :A-colon diverticulysis with abscess formation, s/p Laparoscopic right colectomy and anastomosis on 2021/06/05
- 出院 :A-colon diverticulysis with abscess formation, s/p Laparoscopic right colectomy and anastomosis on 2021/06/05

主訴

- abdominal pain over RLQ for three days.

病史

- The 24-year-old female denied other systemic disease.
  According to the patient, she had suffered from periumbilical abdominal pain since 5/31, and the pain shifted to right lower quadrant on 6/2. Fever was also found. She went to LMD first, where appendicitis couldn't be ruled out. Then, she called at our ER, where PE showed A7 tenderness without rebounding pain. There was no URI symptoms, no N/V, no diarrhea, headache noted. At ER, empirical antibiotics with cefazolin + metronidazole were given. U/A revealed pyuria and bacturia but no symptoms. Abdominal CT revealed A-colon diverticulysis with abscess formation. Emergent surgery of Laparoscopic right colectomy and anastomosis were performed. One penrose drain was placed. After operation, she was admitted for post-op care and antibiotics treatment.

體檢發現

- Anal inspection: fissure 12"
  - General appearance :
    - easy looking , conscious clear , without cardiopulmonary distress
    - Vital signs自動帶入date 體溫(BT) 脈搏(P) 呼吸(R) 血壓(BP) 身高(BH) 體重(BW) BMI
      20210605 37.6C 83/min 13/min 132/83mmHg 165cm 60kg 22.0
    - Vital signs: B.P. 132/83 mmHg , P.R. 83 /min , R.R. 13 /min , B.T. 37.6 ℃
  - HEENT :
    - Eyes: no anemic conjunctiva, no icteric sclera, normal EOM
    - ENT: no otorrhea, no rhinorrhea, no erythematous mucosa, no injected tonsil, no deformity
  - Neck: supple, freely movable, trachea midline,no deformity, no engorged jugular vein , no palpable thyroid or lymph node enlargement
  - Chest:
    - Inspection: Grossly normal, symmetric and fully expansion, no accessory muscle used, no operation scar, no spider angiomata, no gynecomastia
    - Palpation: no palpable mass or nodule, no subcutaneous emphysema, no tenderness
    - Ausculation: clear breathing sound
  - Heart:
    - Percussion: normal heart size
    - Ausculation: regular heart beat, normal S1, S2, no gallop, no murmur
  - Abdomen:
    - Inspection: in

- ▪ flat contour, symetrically, no scar, no stria, no dilated veins, no rashes
    - Ausculation: normoactive bowel sound, no abdominal bruits
    - palpation: soft, no muscle guarding or rigidity, no local tenderness, no rebounding pain, no palpable mass, no palpable pulsation
    - Percussion: no shifting dulllness, no hepatomegaly, no splenomagaly
  -

Peripheral pulsation :	CA	BA	RA	FA	PA	PTA	DPA
Right side :	++	++	++	++	++	++	++/+++
Left side :	++	++	++	++	++	++	++/+++

  - Genito-anus: no tenderness or palpable mass lesion or rectal shelf, normal external genitalia, normal anal tone, brown stool

CRS CONDITION

  -
    - Lung

手術日期及方法

- 手術日期及方法20210605 Laparoscopic right colectomy and anastomosis
  20210605 Laparoscopy

住院治療經過

- Due to A-colon diverticulysis with abscess formation, emergent operation of Laparoscopic right colectomy and anastomosis was done on 2021/06/05. We arranged post-op care at ward. She began to try water on 6/7, blend II diet on 6/9. She complained of intermittent abdominal tenderness over wound, but tolerable. Under stable condition, she was discharged with OPD follow up.

合併症

一般檢查

- **檢驗項目 :Differential Count(D.C),Platelet 檢體:Blood 報告日期 :1100610**

DC: SEG. % (40-74) BAND % (0-5) LYM 13.1 % (19-48)
MONO. 5.5 % (3.4-9) EOSINO. 1.9 % (0-7) BASO. 0.2 % (0-1.5)
META. % (0) MYELO. % (0) PROMYELO. % (0)
BLAST. % (0) GRAN. % Atypical Cell %
ATYP.LYM. % NEUT. 79.3 % (40-74)
NRBC/100WBC. 0.0
PLATELET COUNT. 396 x10^3/μL (150-400)
備註:
Remark ：

檢驗項目 :WBC,RBC,HGB,HCT,MCV 檢體:Blood 報告日期 :1100610

CBC: WBC 9420 /μL (M:3900-10600;F:3500-11000)
RBC 4.10 x10^6/μL (M:4.5-5.9;F:4.0-5.2)
HGB 11.0 g/dL (M:13.5-17.5;F:12-16)
HCT 34.0 % (M:40-52;F:37-47)
MCV 82.9 fL (80.0-100.0)
MCH 26.8 pg (26.0-34.0)
MCHC 32.4 g/dL (31.0-37.0)
RDW 13.2 % (11.5-14.5)
ANC 7470 /uL
(1780~6040/uL)
MPV 9.7 fL (9 -13 fL)
(6歲請參閱兒童血液參考值)
備註:
Remark ：
兒童血液參考值(參考來源：中兒雜誌：167-73.1988)：

Age	RBC(×10^6μL)	Hgb(g/dL)	Hct(%)	MCV(fl)	MCH(pg)	MCHC(g/dL)
≦1day	4.01-4.93	14.2-17.2	43.3-52.3	103.9-110.1	33.9-36.3	32.5-33.3
>Iday-8day	3.99-4.98	14.0-17.4	41.0-51.4	97.4-106.7	33.3-36.1	32.9-34.9
9day-≦6M	2.89-4.83	9.6-12.8	27.3-37.2	74.9-98.8	25.5-33.7	32.9-35.6

- >6M- 6yr 4.28-5.05 11.6-1 3.7 34.2-39.8 74.9-84.6 25.2-29.1 32.6-35.1

Age	WBC(μL)	Neutrophils(%)	Lymphocytes(%)	Monocytes(%)	Eosioophils(%)
≦1day	14300-24900	61.2- 79.6	12.9- 27.7	3.5-9.5	0-3.3
>lday-8day	5200-13400	32.6- 70.7	16.8- 48.1	5.2-1 9.6	1-6.1
9day-≦1yr	5.00-12000	9.4-30.4	55.6-82.6	1.2-11.7	0.1-7.3
>lyr-≦4yr	6700-11800	13.9-49.5	44.7-81.6	1.3-7.2	0-4.3
>4yr-6yr	6000-10400	27.8- 57.6	34.4-62.8	2-7.6	0-6.8

檢驗項目 :GOT,GPT,Albumin 檢體:Blood 報告日期 :1100610

AST(SGOT)	12	U/L	(8 - 38)
ALT(SGPT)	3	U/L	(M:10-50,F:10-35)
LDH		U/L	(120-240)
ALP		U/L	(50-190)
Albumin	3.3	g/dL	(3.5-5.0)
Total Protein		g/dL	(6.0-8.0)
FIB-4			(Metavir fibrosis stage F3 >= 3.25)

備註:
Remark ：

檢驗項目 :BUN,Creatinine,Na,K,Ca 檢體:Blood 報告日期 :1100610

BUN	9	mg/dL	(5- 25)
Creatinine	0.81	mg/dL	(M:0.7-1.4 , F:0.5-0.9)
Na	140	mEq/L	(137-153)
K	4.1	mEq/L	(3.5-5.3)
Cl		mEq/L	(95-105)
Ca	8.3	mg/dL	(8.4-10.2)
eGFR	92.33	ml/min/1.73㎡	(STAGE1: >=90 ; STAGE2: 60-89 ; STAGE3: 30-59.9 ; STAGE4: 15-30 ; STAGE5: 15)

備註:
Remark ：

檢驗項目 :Bilirubin Total,Bilirubin Direct 檢體:Blood 報告日期 :1100610

Total Bilirubin	0.40	mg/dL	(0.2-1.2)
Direct Bilirubin	0.14	mg/dL	(=0.2)(自106年10月20日起修訂Direct Bilirubin參考值)

備註:
Remark ：

特殊檢查

病理報告

- 活體組織切片病理報告 手術日期：**110/06/05** 病理號：**S1018385**

診斷醫師:吳致瑩 報告日期:110/06/09
Pathologic diagnosis:
1. Ascending colon, terminal ileum, and appendix, lapa right hemicolectomy --- Diverticulitis with fecalith impaction and focal active serositis, of ascending colon. The appendix, ileocecal valve and bilateral cut ends are free.
2. IC root, resection --- Unremarkable fibrovascular tissue and lymph nodes (0/8).

Gross description:
The specimen consists of 1) a segment of colon, 9 cm in length, and 4.5 cm in proximal circumference, 3.5 cm in distal cut end circumference, and a segment of terminal ileum, 3.5 cm in length, and 2 cm in cut end circumference. An unremarkable appendix, 6.5x0.5 cm, is noted. There is an out-pouching lesion, 3x2.2 cm, noted in the ascneding colon, 3 cm to the nearest cut end. Some yellow fibrinoid exudate is noted on the serosa. On section, no mass lesion is identified grossly. The appendix, ileocecal valve, and both cut ends are free. 2) "IC root", a piece, 1.6x1.5x0.5 cm. Representative part for section: A) proximal cut end B) distal cut end C) ileocecal valve D) appendix E-H) lesion J) specimen 2.
#T-59420_2 #M-40630_2 0 1105 000000

- #T-C4560_2 #M-09183_2 0 1105 000000
  #T-59200_2 #M-09183_2 0 1105 000000

其它

出院時情況

- 3.改門診治療

出院指示

-

藥名	劑量	劑量單位	用法	頻次	數量	數量單位	現況	天數	單位
Voren-K tab 25mg	1	TAB	PO	TID	21	TAB	TH	7	天
Pantoloc tab 40mg	1	TAB	PO	QDAC	7	TAB	TH	7	天

出院計劃

主治醫師 2222H 林俊余 / 住院醫師 2343L 張嘉育

正式摘要(**電子簽章：000000218847042)**

W85 / W85

doctorRank:B
